# Effectiveness and implementation outcomes of the multimodal strategy in IPC for pediatric ventilator-associated events at a provincial hospital in Vietnam: a hybrid II implementation design

**DOI:** 10.1017/ash.2025.80

**Published:** 2025-09-03

**Authors:** Dang Thi Thu Huong, Tran Minh Dien, Pham Duy Hien, Nguyen Van Dat, Le Nho Khue, Phan Van Tuong, Anh Thi-Kim Le

**Affiliations:** 1 VietNam National Children’s Hospital, Hanoi; 2 Bac Ninh Obstetrics and pediatrics Hospital; 3 Hanoi University of Public Health

**Keywords:** infection prevention and control, infection prevention and control assessment framework (IPCAF), pediatric ventilator-associated events (Ped-VAE), multimodal strategy

## Abstract

**Background:** Vietnam has the national guidelines for infection control in hospitals and it also recommends the application of WHO’s IPCAF framework to do self-evaluation of infection prevention and control (IPC) activities and plan to improve them in hospitals. **Objective:** Our study aimed to implement the multimodal strategy for IPC, in which our expected outcome was the practices of doctors and nurses for pediatric ventilator-associated events (Ped-VAE). **Design:** We used the implementation research approach with the hybrid design of quasi-experimental pre-post comparison without control group. All 16 doctors and 32 nurses at the Department of PICU were observed 3 times that practicing the IPC packages for PedVAE guided by MOH. The implementation strategies used included Plan, Restructure, Train, and Quality Management. **Results:** Four over six steps practised by doctors and 5/10 steps practised by nurses for PedVAE were well practised after the intervention with significantly higher proportion of right practices (p<0.001). The practices of doctors had insignificant changes between pre-post intervention, including hand hygiene (85.4% and 83.3% of right practice at pre-post intervention, respectively) and daily assessment of weaning from mechanical ventilation (54.2%-68.7%). Most unchanged practices among nurses were steps of ensuring humidification and heating of inhaled gas for in patients with artificial airway.

All practice scores of the whole steps among doctors and nurses had statistically significant increase after intervention. Our implementation strategies were highly assessed by providers (doctors and nurses) and hospital managers in terms of the its acceptibility, feasibility and sustainability. **Conclusion:** The implementation of multimodal strategy in IPC for pediatric ventilator-associated events is effective and acceptable and feasible for hospitals at city/province level in Vietnam. In addition with improving practices of healthcare staffs, hospitals should regularly assess and upgrade ventilators machines to ensure the effectiveness of IPC.

